# Research progress of exosomes used in the Alzheimer's disease treatment

**DOI:** 10.1186/s11671-025-04361-0

**Published:** 2025-10-01

**Authors:** Xiaoqin Gao, Ke Yang, Xiaokui Yuan, Mengyuan Song, Tong Wang, Chenlan Shen

**Affiliations:** 1 Department of Clinical Laboratory, People’s Hospital of Dayi County, Chengdu, 611330 China; 2 Department of Laboratory Medicine, Clinical Laboratory Medicine Research Center, West China Hospital, Sichuan University, Sichuan Clinical Research Center for Laboratory Medicine, Chengdu, 610041 China; 3 Department of Laboratory Medicine, Chengdu Shangjin Nanfu Hospital, Chengdu, 610000 China; 4 The Fourth People’s Hospital of Chengdu, The Clinical Hospital of Chengdu Brain Science Institute, University of Electronic Science and Technology of China, Chengdu, 610036 China

**Keywords:** Alzheimer’s disease, Exosomes, Exosome-based nano-therapeutic, Molecular mechanism

## Abstract

**Abstract:**

Alzheimer's disease (AD) is a common form of dementia characterized by memory loss, cognitive and linguistic abilities declining and self-care capabilities diminishment. With the aging population globally, AD poses a significant threat to public health. Current treatments for AD aim to alleviate symptoms and slow down disease progression, but due to the limited understanding of underlying disease mechanisms, AD is still impossible to be cured yet. In recent years, there has been an exponential growth in exosome-related research because of their excellent biocompatibility ability, loading capacity and cellular internalization, making exosome to be one of the hotspots and a promising strategy in AD therapy research. This comprehensive review systematically explores the potential roles of various exosome-based nanotherapeutic strategy in AD treatment, with a particular focus on their specific biological mechanisms of action. Firstly, we elaborated on the pathological mechanisms of AD formation as well as the mechanisms related to the formation, secretion and function of exosome. Additionally, we highlighted the research progress in the development of exosome-based nanotherapeutic strategies for AD treatment and their corresponding biological mechanisms. Furthermore, we delved into the challenges and opportunities these strategies facing in clinical application. Looking forward to future research directions and trends, our review aims to provide a more comprehensive understanding and guidance with the application of exosome in AD treatment. Exosome-based nanotherapeutic strategies, as a new therapeutic approach, have opened up new possibilities for the treatment of AD and brought new light to patients.

**Graphical abstract:**

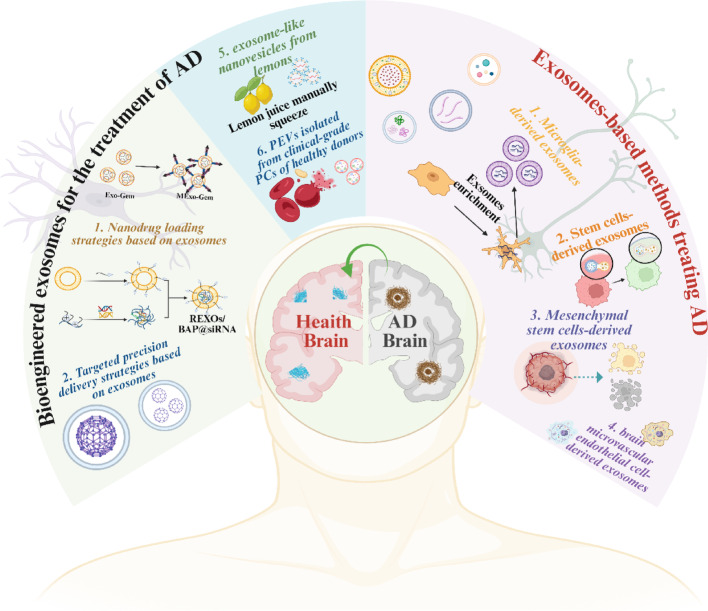
Schematic diagram of exosome-based nanotherapy strategies for the treatment of AD. It can be roughly classified as: exosomes-based methods treating AD and bioengineered exosomes for the treatment of AD.

## Introduction

Alzheimer's disease (AD) is one of the most prevalent neurodegenerative disorders. It is expected that there will be 152 million AD cases worldwide by 2050 due to population aging [1]. In terms of clinical manifestation, AD is characterized by memory and cognition decline, with its etiology yet to be fully elucidated [2]. At present, the widely accepted hypotheses of the pathogenesis of AD mainly include amyloid protein deposition, abnormal phosphorylation of Tau protein, mitochondrial dysfunction, oxidative stress, etc. [2]. Among these assumptions, amyloid deposition hypothesis is one of the most concerned one at present. This hypothesis suggests that the main cause of AD is the abnormal deposition of amyloid beta proteins (Aβ) forming plaques in the brain, which is thought to be toxic to neurons, resulting in neuronal damage and death. Clinically, the treatment for AD mainly targets symptom management rather than the pathological processes of neurodegeneration. So far, the U.S. Food and Drug Administration (FDA) has approved only seven drugs/drug combinations, including Aducanumab, Donepezil, Galantamine, Livastine, Memantine, Lyca-resistant mab and the combination of Memantine and Donepezil [3], which can only partially improve symptoms of AD and are limited by higher costs and more side effects. In addition, the onset and progression of AD is latent, and by the time most patients seek medical attention, neurons have been lost for decades, and drug therapy can only be used for patients in the early and middle stages, so it is unlikely that most patients in the middle and late stages will achieve the desired effect through the corresponding drug treatment [4]. Surgical treatment (deep brain electrode stimulation) can also improve cognitive function in some AD patients, but surgical risks and high costs limit its wide application in clinical practice [5, 6]. Therefore, new treatment strategies for AD are urgently needed to meet clinical needs.

**Fig. 1:**
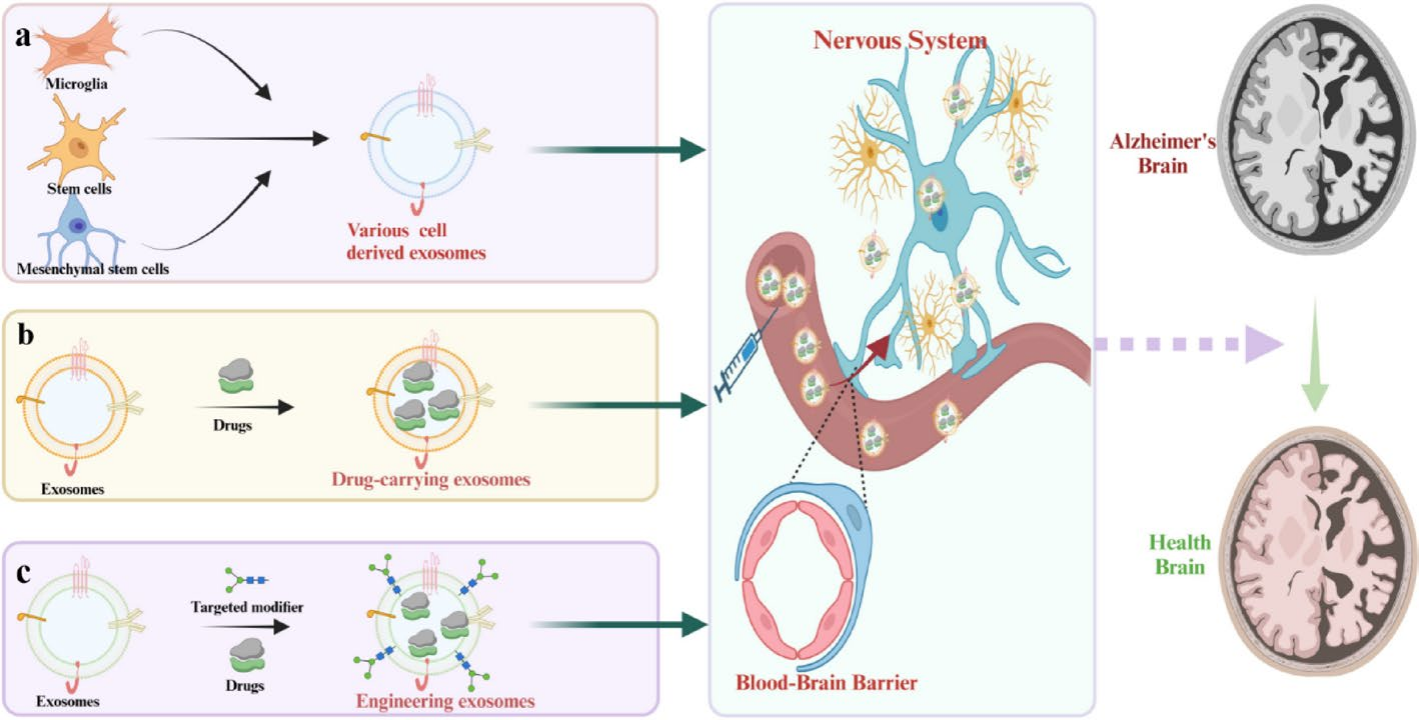
Schematic diagram of exosome-based nanotherapeutic strategies for the treatment of AD. It can be roughly classified as: **a** exosomes of various nerve cells are used to improve AD. **b** Exosomes used as load-related therapeutics to improve AD. **c** Exosomes should be engineered to have the ability to target nerve cells and load therapeutic drugs

**Fig. 2:**
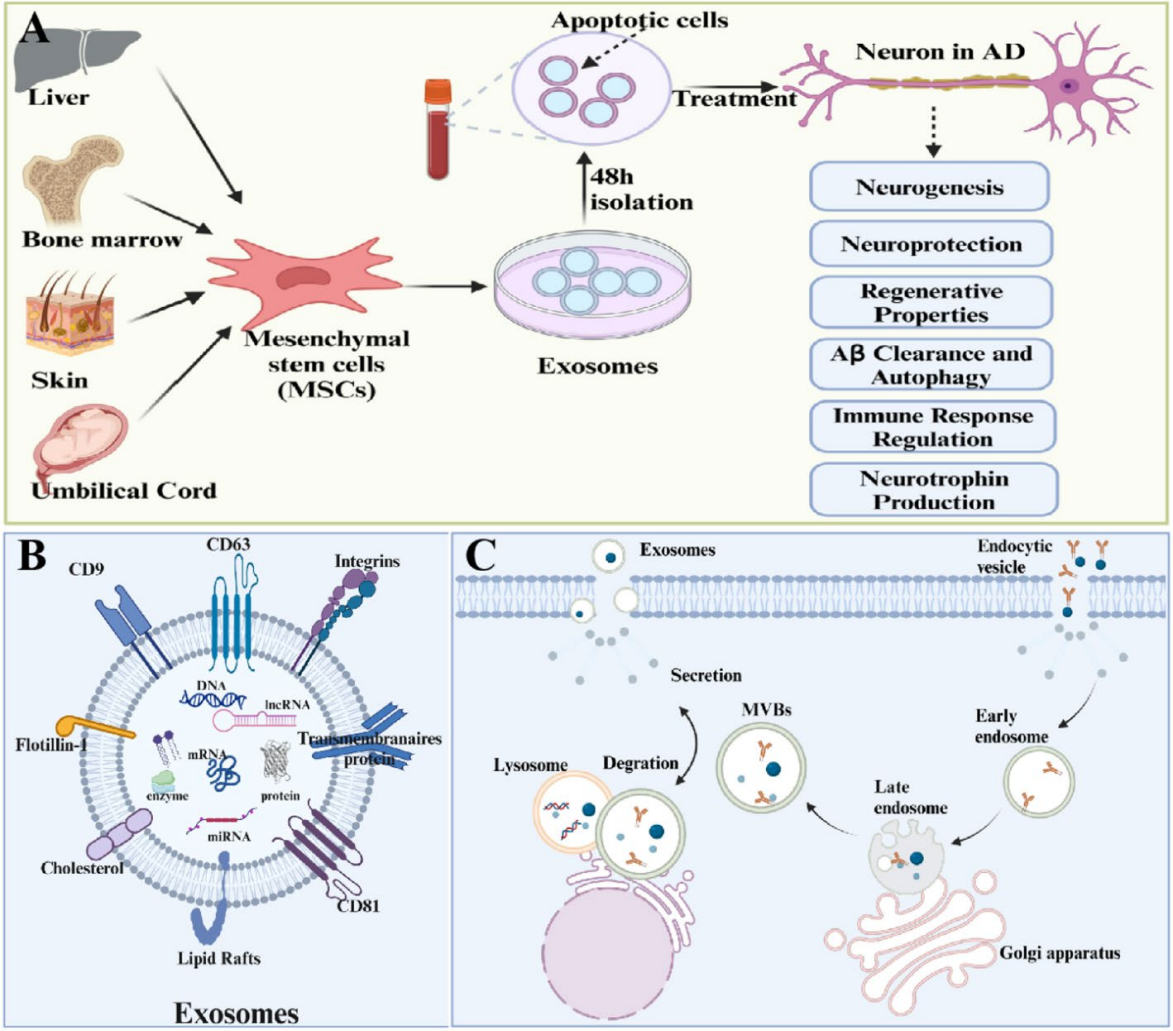
Schematic illustration of different sources of mesenchymal stem cells and their properties of importance in AD treatment along with exosomes (**A**). Biogenetic process of exosomes (**B**) and composition of exosomes (**C**) [16]. Schematic diagram of possible pathologic mechanisms in the development and progression of AD (**D**)

**Fig. 3:**
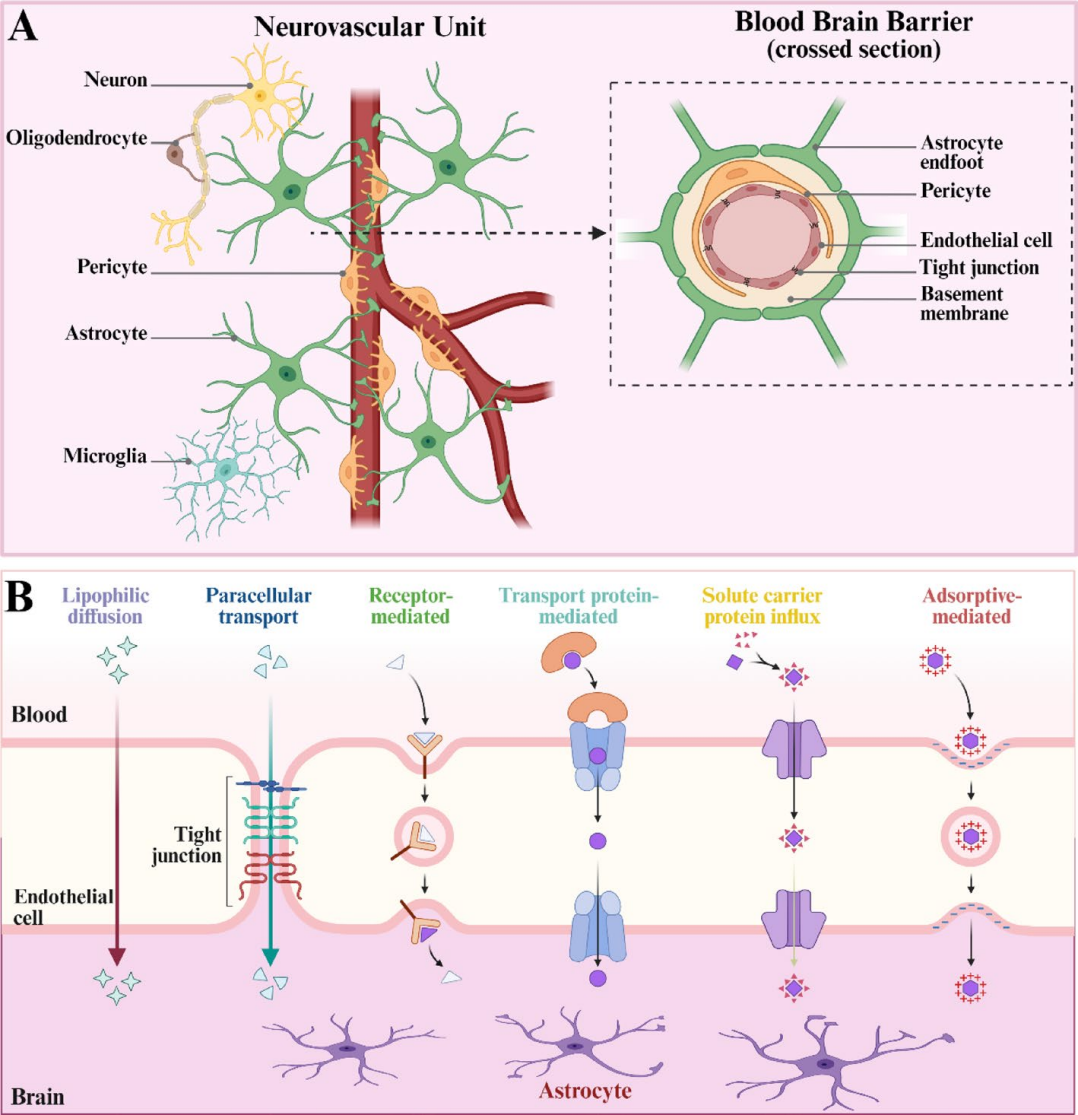
Schematic diagram of the biological structure of the blood–brain barrier. **A** The biological composition of the blood–brain barrier. **B** The mechanism of substance transport in the blood–brain barrier under physiological conditions

In recent year, researchers have used a variety of methods (such as monoclonal antibodies, inhibitors, antagonists, etc.) to develop effective AD treatments to improve patients' cognitive function and life quality [7]. However, these drugs are not targeted and have significant side effects. Besides, with the advancement of nanotechnology, it is possible to achieve more precise treatment by using nanoparticles (NPs) (such as MOFs, nano-polymers, liposomes, etc.) to deliver therapeutic drugs to the lesion site. However, these nanomaterials may trigger an immune response and their delivery targeting accuracy is not high [8]. It is noteworthy that exosomes are tiny vesicles about 30-150 nm in diameter that are formed by the formation of endosomes, containing biomolecules such as proteins, RNAs and lipids, which can effectively evade the mononuclear phagocytosis system [9]. Besides, exosomes used for therapeutic purposes are mainly derived from human cell lines, containing several unique advantages compared to NPs of non-human origin [10]. For example, (1) exosomes generally exhibit the same tropism as their parent cells [11–14], a property that facilitates for their research and development as therapeutic agents. For instance, macrophages are key immune cells in the body that are naturally recruited to inflammation-prone sites (Fig. 1). Moreover, exosomes derived from macrophages may accumulate in the chronically inflamed brains of patients with neurodegenerative diseases due to the inflammation-guided chemotaxis (Fig. 2A) [15, 16]. Additionally, Nisim et al. also found that exosomes derived from mesenchymal stem cells could target and accumulate in certain regions of the brains of pathological-related mouse model [17]. Biocompatibility can greatly reduce drug side effects and increase their retention time in the human circulatory system. Membrane surface programming provides great possibilities for drug loading and exosome engineering editing [11, 12]. In addition, the blood–brain barrier (BBB), as the physiological barrier formed by the blood vessels, has the unique property of precisely controlling the molecules that enter the central nervous system (CNS), which considered to be one of the obstacles to effective treatment of AD (Fig. 3). However, exosomes can efficiently cross the BBB for either targeted or untargeted drug delivery, thereby reducing the dose required to achieve therapeutic effects [12].

In this study, we systematically investigated the potential therapeutic roles of exosome-based nanotherapeutic strategies in the AD treatment, with a particular focus on the specific biological mechanisms underlying their efficacy. Initially, we delineated the pathophysiological mechanisms underlying the formation of AD. Subsequently, we provided a detailed account of the mechanisms related to the biogenesis, secretion and functions of exosomes. Furthermore, we summarized the research progresses in the application of exosomes for the AD treatment, as well as the development of exosome-based nanotherapeutic strategies and their underlying biological mechanisms. Finally, we explored the challenges and opportunities associated with their clinical application. Looking forward to future research directions and trends, this comprehensive analysis aims to provide a more in-depth understanding and guidance for the application of exosomes in the AD treatment.

## An overview of the pathogenic mechanism of Alzheimer's disease

**Fig. 4:**
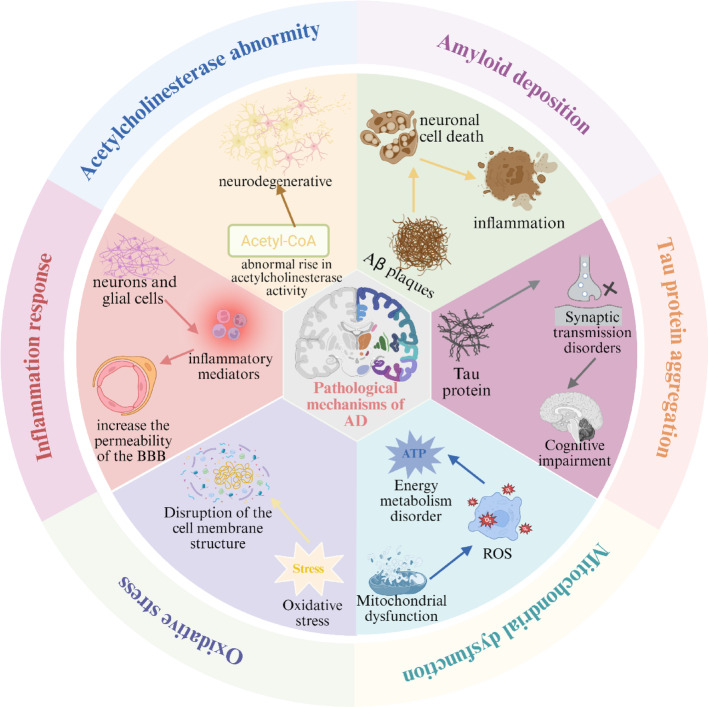
Schematic diagram of possible pathologic mechanisms in the development and progression of AD

Alzheimer's disease, commonly referred to be senile dementia, is a chronic neurodegenerative disorder characterized by progressive cognitive decline, predominantly affecting the cognitive faculties of the elderly [18]. The etiology of AD remains to be elucidated [19]. Nonetheless, based on extant theories, the putative pathogenic mechanisms are as follows (Fig. 4).

### Amyloid deposition

The amyloid precursor protein (APP) is a transmembrane protein present on the surface of neurons and other cell membranes [20]. Its metabolic pathway involves various proteases, including α-secretase, β-secretase (BACE1) and γ-secretase [21]. In normal physiological processes, APP is predominantly cleaved via the α-secretase pathway, yielding a soluble extracellular fragment (sAPPα) and an intracellular fragment, a process that does not generate Aβ [22]. However, in the pathophysiology of AD, APP is also cleaved via the β-secretase pathway, producing a larger membrane-bound fragment (C99) and a smaller soluble extracellular fragment (sAPPβ) [23]. Then, C99 is subsequently cleaved by the γ-secretase complex, which is a multiprotein complex tasked with cleaving the transmembrane domain of APP, releasing Aβ and an intracellular domain (AICD) [24]. The cleavage sites of the γ-secretase are variable, producing Aβ of different lengths, among which Aβ42 is the most amyloidogenic form [25]. Finally, the formation of amyloid plaques is a complex multistep process involving the generation, aggregation and deposition of Aβ, which in turn interferes with synaptic function and elicits neuronal damage. Subsequently, the resulting Aβ is released outside the cell, accumulating in brain tissue and forming oligomers and fibers through self-assembly. As Aβ continues to accumulate they will further aggregate to form larger plaques called amyloid plaques [26]. Moreover, pathological states such as oxidative stress and inflammation may also facilitate Aβ aggregation and amyloid plaques formation [27]. Certain genetic variants, including mutations in the APP and BACE1 genes, can increase Aβ production or decrease its clearance [28, 29].

### Tau protein aggregation

Tau protein, as a microtubule-associated protein, is predominantly found within the axons of neurons [30]. Its main function is to promote and maintain the stability of microtubules, promote the formation of neuronal cytoskeleton and thus support the morphology and transport of neurons [31]. Under normal physiological conditions, Tau protein can be phosphorylated by various kinases, a reversible process that is essential for its proper function [32]. However, in neurodegenerative diseases such as AD, the phosphorylation of Tau protein is dysregulated, leading to hyperphosphorylation, which in turn compromises its ability to bind with microtubules and maintain their stability. Then, hyperphosphorylated Tau protein begins to aggregate and forms paired helical filaments (PHFs), the primary constituent of neurofibrillary tangles in AD [33]. These PHFs further aggregate into larger structures called Tau tangles, which become entangled within neurons, impeding normal neuronal function and axoplasmic transport [34]. It's worth noting that Tau protein undergoes alternative splicing in different neurons to produce various isoforms, and abnormal splicing can lead to an increase in the proportion of harmful isomers, thus promoting the formation of tangles [35]. Besides, certain genetic mutations, such as those in the microtubule-associated protein tau (MAPT) gene, can directly affect the structure and function of Tau protein, making it more likely to form tangles [36]. Moreover, an imbalance in intracellular calcium concentrations can lead to abnormal phosphorylation of Tau protein, thereby promoting tangles aggregation.

### Mitochondrial dysfunction

Mitochondria, often considered as the powerhouses of the cell, is responsible for generating adenosine triphosphate (ATP), which is the currency of cellular energy. Additionally, mitochondria is instrumental in regulating intracellular calcium levels, maintaining redox balance and facilitating apoptosis [37]. In AD, mitochondrial energy metabolism is disturbed, resulting in diminished production of ATP. This impairment may be associated with the dysfunction of the mitochondrial respiratory chain complexes, especially the reduced activity of Complex I and Complex III [38]. Dysfunctional energy metabolism impedes the ability of neurons to maintain normal physiological activities, thereby impacting cognitive function. Besides, mitochondria are the main source of reactive oxygen species (ROS), which can damage mitochondrial DNA (mtDNA), proteins and lipids, exacerbating mitochondrial dysfunction [37, 39]. Among them, mtDNA is more susceptible to ROS-induced damage than nuclear DNA, and such damage can impair the synthesis of respiratory chain proteins, affecting energy metabolism [40]. In addition, the accumulation of mutations in mtDNA may also lead to mitochondrial dysfunction, as these mutations can compromise the function of respiratory chain components encoded by mitochondrial genes. Moreover, mitophagy, the process by which cells clear damaged or dysfunctional mitochondria, plays a crucial role [41]. Under normal circumstances, cells possess an antioxidant system to neutralize these radicals. However, in the course of AD, the mitochondrial antioxidant defenses are disrupted, leading to an increased generation and decreased clearance of ROS. As the result, the impairment of mitophagy can result in the accumulation of damaged mitochondria, further intensifying mitochondrial dysfunction and promoting the progression of AD [41].

### Oxidative stress

Under normal physiological conditions, mitochondria, endoplasmic reticulum and other cellular components generate a low level of ROS, including superoxide anion (O_2_-), hydrogen peroxide (H_2_O_2_) and hydroxyl radical (OH^·^) [42]. In the brains of AD patients, the production of ROS may be augmented due to mitochondrial dysfunction, enzymatic anomalies or inflammatory responses. In addition, as the organism's antioxidant capacity is diminished, ROS is not unable to be removed effectively. Then, excess ROS can oxidize amino acid residues in proteins, leading to structural alteration, function impairment as well as DNA damage [43]. Then, oxidatively damaged proteins can result in protein aggregation and dysfunction within neurons [42]. It's worth noting that mitochondrial DNA is more vulnerable to ROS attack compared to nuclear DNA in AD, leading to mitochondrial dysfunction and energy metabolic impairment. In addition to its effects on proteins and nucleic acids, ROS can also target polyunsaturated fatty acids in cell membranes, triggering lipid peroxidation [44, 45], which can generate lipid peroxides and free radicals that disrupt the structure and function of cell membranes and perpetuate a deleterious positive feedback loop, thereby exacerbating the progression of AD.

### Inflammation response

In the context of AD, neurons and glial cells elicit a series of inflammatory mediators, which can not only augment inflammatory responses, impair neurons directly but also increase the permeability of the BBB, thus promoting the invasion of peripheral immune system cells. Firstly, the accumulation of Aβ can activate microglia, transitioning them into a pro-inflammatory phenotype. Then, the activated microglia releases pro-inflammatory cytokines (such as TNF-α, IL-1β, and IL-6) and ROS [46, 47]. Moreover, when the complement system is activated, it generates membrane attack complexes (MACs) that can damage neurons and glial cells, thereby exacerbating the inflammatory response [48]. Additionally, peripheral immune system cells, including T cells and B cells, traverse the BBB to enter the brain, where they secrete inflammatory cytokines and cytotoxic molecules, further intensifying neuroinflammation [49].

### Acetylcholinesterase abnormity

Acetylcholinesterase is an enzymatic entity resident within the synaptic cleft and its primary function is to catalyze the rapid degradation of the neurotransmitter acetylcholine (ACh) [50]. Under physiological conditions, acetylcholinesterase activity ensures the rapid clearance of ACh after synthesis, thus terminating the transmission of neural signals. Additionally, acetylcholinesterase is able to bind to Aβ to form complex that not only enhances the stability of the enzyme, but may also modulate its catalytic activity [51, 52]. However, the interaction between Aβ and acetylcholinesterase could potentially diminish the hydrolytic action of the enzyme on ACh via steric hindrance [53]. In the pathological context of AD, this binding may lead to an abnormal rise in acetylcholinesterase activity. So, the excessive acetylcholinesterase activity combined with the decrease of ACh level can promote neurodegenerative changes, thereby aggravating the pathological progress of AD [54]. Besides, the decreased ACh level can lead to synaptic structural damage, encompassing both presynaptic and postsynaptic alterations, which impair the transmission of neural signals and compromises the stability of the neural network. Based on this mechanism, the inhibition of acetylcholinesterase is an efficacious therapeutic approach for AD [55]. Therefore, medications that can reduce the activity of acetylcholinesterase, such as Donepezil and Rivastigmine, can be used to increase the level of ACh in the synaptic cleft and improve cognitive function in AD patients [56–60].

## The blood–brain barrier (BBB)

The BBB is a dynamic and selective permeable structure within the central nervous system (CNS), composed of vascular endothelial cells, pericytes, astrocyte foot processes, and basal membranes [61]. It precisely regulates the exchange of substances between the blood and brain tissue, maintaining the homeostasis of the brain environment and protecting nerve cells from harmful substances from the periphery. Its core characteristics include a continuous and pore-free barrier formed by tight junctions between capillary endothelial cells, as well as a highly specialized transport system [62].

The BBB is mainly composed of brain capillary endothelial cells. The cells are separated by tight junction proteins (such as occludin, claudin-5), forming a physical barrier that effectively blocks the paracellular diffusion of macromolecules and polar substances [63]. The basement membrane of endothelial cells is composed of laminin and collagen. Pericyte parts wrap around the endothelial cells and participate in vascular regulation. The foot processes of astrocytes dynamically affect the permeability of the barrier by secreting neurotrophic factors and regulating ion balance. In addition, microglia and neuronal terminals also participate in the regulation of the microenvironment of barrier function through paracrine signals [64].

The function of BBB has multiple protective and selective properties. It allows small lipid-soluble substances (such as oxygen and carbon dioxide) to freely diffuse, while essential nutrients such as glucose and amino acids need to be transported actively through specific transporters [65]. Through efflux transporters (such as P-glycoprotein), lipophilic drugs and metabolic wastes are pumped back into the blood to reduce the accumulation of toxins in the brain [66]. At the same time, BBB can restrict the entry of immune cells and inflammatory factors, avoiding excessive immune responses in the CNS [67].

In the field of drug delivery, the high selectivity of the BBB has become a major obstacle in the treatment of central nervous system diseases [68]. Current research focuses on strategies to overcome the BBB, including receptor-mediated transport (such as transferrin receptor targeting), temporary opening of the barrier (such as ultrasound combined with microbubble technology), and nanocarrier delivery systems [69]. Additionally, inhibitors targeting efflux transporters (such as verapamil) can enhance the brain distribution of drugs, providing a new research direction for treating diseases such as AD and glioma [70].

## An overview of exosomes

In recent years, the role of exosomes in neurodevelopmental disorders (NDs) has attracted great interest due to their low immunogenicity, biocompatibility, special spherical load structure and ability to penetrate the BBB [71–77].

### Formation and composition of exosomes

The generation of exosomes is depicted in (Fig. 2B, C) [74]. Specifically, the plasma membrane first invaginates to form early sorting endosomes (ESEs), which contain extracellular soluble proteins and surface proteins [75]. Concurrently, inward budding of the plasma membrane can fuse with pre-existing ESEs. Subsequently, these ESEs mature into late sorting endosomes (LSEs), with their membranes invaginating once again to encapsulate intracellular contents, yielding multiple intraluminal vesicles (ILVs) of varying sizes and compositions, ultimately forming multivesicular bodies (MVBs) [76]. Finally, MVBs can be degraded by fusion with autophagosomes or lysosomes, or by fusion with the plasma membrane to release ILVs in the form of exosomes [77]. Furthermore, the biogenesis, cargo sorting and secretion of exosomes are regulated by various proteins, such as ESCRT, Alix, CD81, CD9, CD63, TSG101, and others [74, 77, 78]. Finally, the release of exosomes involves the participation of soluble N-ethylmaleimide-sensitive factor attachment receptor (SNARE) complexes and the synaptosome-associated protein family [79]. Consequently, exosomes derived from different parental cells all contain certain common protein markers, including Alix, TSG101, SNARE and RAB GTPases [80]. Moreover, certain lipid components, such as cholesterol, ceramides and sphingomyelin, also actively promote the formation and release of exosomes [76].

During the formation of exosomes, a variety of molecules, including lipids, proteins, metabolites, nucleic acids and cytoplasm, are actively or passively loaded into the vesicles [79]. The content varies depending on the type of parental cell [80]. Since exosomes originate from the plasma membrane, their lipid components, such as cholesterol, phospholipids and phosphatidylethanolamine, are primarily distributed on their surface, which are similar to the composition of the phospholipid bilayer of parental cells [80]. Additionally, with the advancement of omics technologies and bioinformatics, an increasing number of exosomes components have been identified in recent years [81, 82].

### The characteristics of exosomes

Exosomes represent promising carriers for biomolecules/drugs, as they can protect cargoes from enzymatic degradation or macrophage clearance and even enhance their biological functions [78]. Furthermore, they also can efficiently mediate intercellular communication and regulate cellular activities [75]. Specifically, exosomes mediate intercellular communication through three main mechanisms: (1) direct interaction between exosome membrane proteins and target membrane proteins. Specifically, the membrane proteins on the surface of exosomes can directly bind to the membrane proteins on the surface of target cells, thereby activating the signaling pathway within the target cells. This combination can precisely regulate cellular communication, but it is also limited in space and time. (2) The enzymatic cleavage fragments of exosome membrane proteins serve as ligands. To be specific, exosome membrane proteins are cleaved by proteases in the extracellular matrix and the resulting fragments can be used as ligands to bind to receptors on the surface of target cells and activate intracellular signaling pathways, which can increase the diversity of communication between cells. However, the enzymolysis process of exosome membrane proteins is affected by many factors, and the accuracy of signal transmission may be affected. (3) The fusion of exosome membrane and target membrane. Exosome membrane can directly fuse with the target cell membrane to transfer proteins, RNA and other molecules in exosomes to target cells, thereby regulating the biological activity of target cells [74, 75, 83]. Taken together, the excellent loading capacity and unique cellular internalization process give exosomes unique advantages as potential AD therapeutic agents/carriers [84].

## Exosomes-based methods treating AD

### Microglia-derived exosomes

**Fig. 5:**
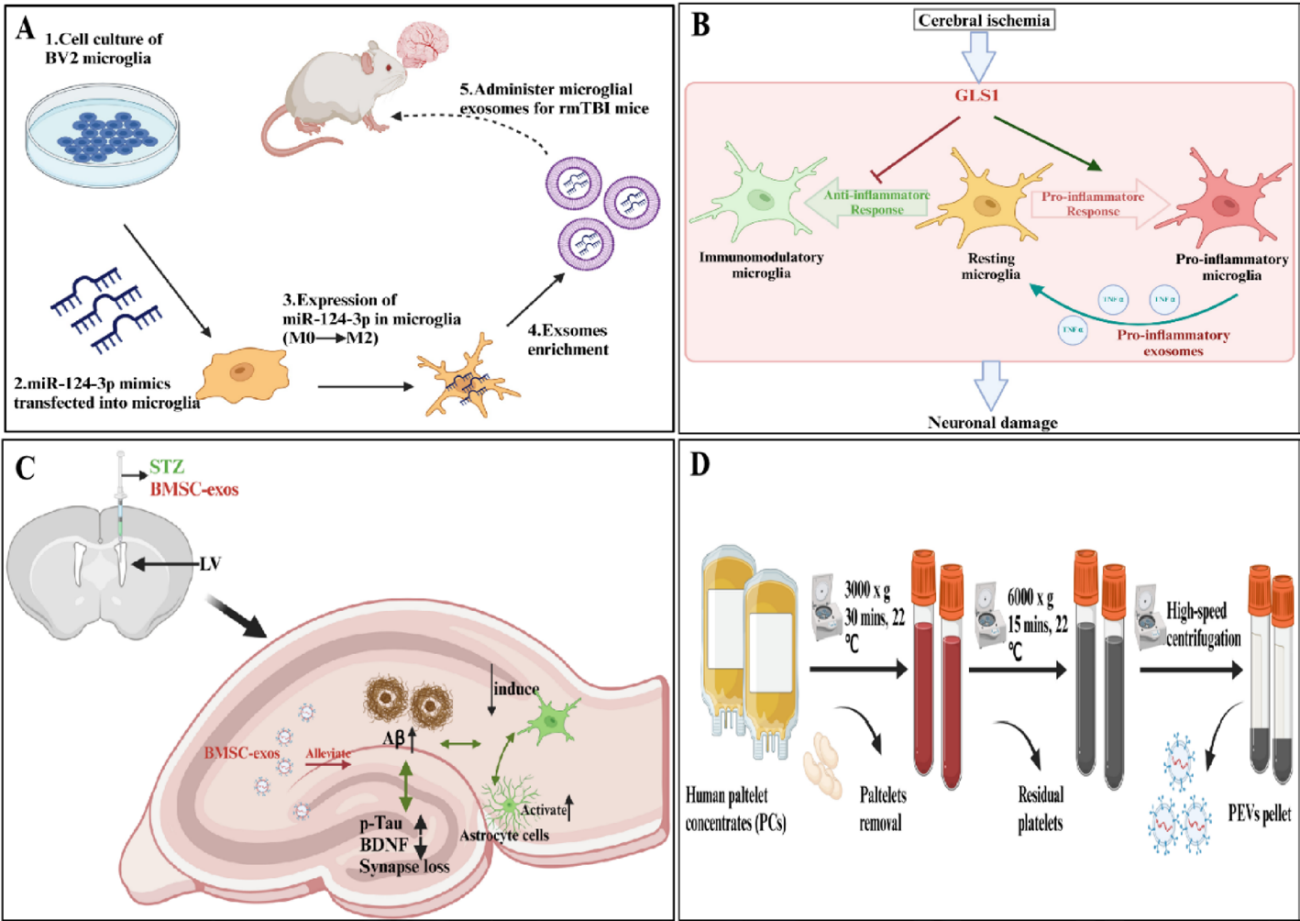
Schematic of in vivo experiments of microglia exosome miR-124-3p to reduce neurodegeneration and improve cognitive outcomes [87] (**A**). Schematic illustration of glutaminase 1 modulating neuroinflammation after cerebral ischemia by enhancing microglial activation and pro-inflammatory exosome release [88] (**B**). Exosomes derived from bone-marrow mesenchymal stem cells alleviate neuroinflammation and synaptic damage by inhibiting the activation of glial cells in the hippocampus [99] (**C**). Diagram illustrating the process of preparing PEVs [102] (**D**)

Microglia are the resident immune cells of the brain, playing a complex role in neurodegenerative diseases, including inflammation regulation, neuroprotection, and the clearance of cellular debris. Microglia-derived exosomes have also been found to play a role in the treatment of AD. Li et al. discovered that exosomes from M2-polarized microglia (M2-EXOs) could exert a protective effect in the pathogenesis of AD by improving PINK1/Parkin-mediated mitochondrial autophagy [85]. Similarly, exosomes from M2 microglia modulated by 1070 nm light could alleviate the β-amyloid burden and improve cognitive function in an AD mouse model by reducing neuroinflammation and promoting neuronal dendritic spine plasticity [86]. Moreover, the content of miR-124-3p in exosomes from microglia after repetitive mild traumatic brain injury (rmTBI) (EXO-124) increased and alleviated neurodegeneration by targeting the Rela/ApoE signaling pathway (Fig. 5A) [87]. Additionally, glutaminase 1 could enhance microglial activation and the release of pro-inflammatory exosomes to regulate neuroinflammation post-cerebral ischemia (Fig. 5B) [88]. All the above reports have revealed the possibility of using exosomes derived from small stromal cells in the treatment of AD.

### Stem cells-derived exosomes

Stem cells-derived exosomes (NSC-exos) contain various biomolecules, such as proteins, RNAs and lipids, can act as intercellular messengers involved in cell communication processes. In the treatment of AD, NSC-exos can carry beneficial biomolecules for neural cells, helping to improve or repair neural cell function (as summarized in Table 1). A meta-analysis has shown that NSC-exos demonstrate significant efficacy in treating AD models. Mechanistically, NSC-exos treatment significantly reduced Aβ plaques in the hippocampus and lowered pro-inflammatory cytokines tumor necrosis factor α (TNFα) and interleukin-1β (IL-1β) [89]. Besides, it can also downregulate the NF-B/ERK/JNK-related signaling pathways in glioma cell line HMC3 (ATCC-CRL-3304) and reduce inflammatory mediators associated with neuroinflammation, such as iNOS, IL-1β, TNF-α and IL-6 [90]. In addition, exosomes from other special sources have been reported to have similar functions. For example, (1) heat shock induced NSC-exos have a significant neuroprotective effect on oxidative stress and amyloid β-induced neurotoxicity [91]. (2) Adipose-derived NSC-exos reduced β-amyloid pathology and neuronal apoptosis in AD mouse models and can decrease the levels of apoptosis-related molecules p53, Bax, precursor caspase-3, cleaved caspase-3 and Bcl-2 proteins [92]. (3) Hypoxic-pretreated adipose-derived stem cell exosomes can also improve the cognitive function of AD mouse models by transmitting circ-Epc1 and altering microglia M1/M2 polarization [93]. (4) human non-restrictive somatic stem cell (hUSSC)-derived exosomes can reduce Aβ accumulation to improve learning and memory function as well as up-regulate synaptic and postsynaptic molecules associated with neuroplasticity, including NMDAR1, integrin β1, synaptophysin, PPKα and GAP-43 [94]. These studies proved that NSC-exos had the potential to treat AD, exploring its specific mechanism and pointing out the direction for further clinical research. However, the development of therapeutic NSC-exos is still in its infancy, and we still need more research to fill out the toolbox for AD therapy.

**Table 1:** Summary of sources, therapeutic targets and molecular mechanisms of NSC-exosomes

Name	Sources	Therapeutic targets	Molecular mechanisms	References
NSC-exos	ATCC-BYS012	Human neuroblastoma cells SH-SY5Y	NSC-exos controlled the associated molecular processes to drastically lower Aβ and p-tau. A dose dependent reduction in β- and γ-secretase, acetylcholinesterase, GSK3β, CDK5, and activated α-secretase activities was also seen	[90]
HS-derived EVs	Rat neural stem cells	Apoptosis and oxidative stress	HS-derived EVs exhibited greater neuroprotection against not only oxidative stress but also Aβ induced neurotoxicity compared to NHS-derived EVs	[91]
ADSC-Exo	Adipose-derived stem cells (ADSC)	AD neuronal cells	ADSC-Exo can be a therapeutic source to ameliorate the progression of Aβ-induced neuronal death and AD	[92]
ADSC exosomes	Hypoxic pretreated adipose-derived stem cells (ADSCs)	Hippocampal microglia	Hypoxic pretreatment of ADSC exosomes improved cognition by delivery of circ-Epc1 and by shifting microglial M1/M2 polarization in an AD mouse model	[93]
hUSSCs-Exo	hUSSCs	Neuroplasticity proteins	Intranasal administration of hUSSC-derived exosomes could decrease Aβ accumulation and improve learning and memory in the Morris water maze test	[94]
MSCs-exo	Mesenchymal stem cell	Gut microbiota	MSCs-exo can be used for treating AD by promoting Aβ degradation, modulating immune responses, protecting neurology, promoting axonal growth, and improving cognitive impairment	[95]
PC-MSCs-exo	Hypoxia-preconditioned MSCs	Astrocytes and Microglia	PC-MSCs-exo could rescue cognition and memory impairment according to results of the Morris water maze test, reduced plaque deposition, and Aβ levels in the brain; could decrease the activation of astrocytes and microglia	[97]
HucMSC-derived exosomes	hucMSCs	SH-S5Y5 cells	HucMSC-derived exosomes effectively protected SH-S5Y5 cells from Aβ1-40 -induced damage	[98]
BMSC-exos	Bone-marrow mesenchymal stem cells	Microglia and Astrocytes	The hyperactivation of microglia and astrocytes in the hippocampus of the model mice was inhibited after treatment with BMSC-exos via lateral ventricle administration, accompanied by the reduced expression of IL-1β, IL-6, TNF-α, Aβ1-42, and p-Tau and upregulated protein expression of synapse-related proteins and BDNF	[99]
BM-MSC exosomes	Rat bone marrow mesenchymal stem cells	Anti-apoptotic, anti-necrotic and anti-oxidant	The early passage derived exosomes protected neurons through anti-apoptotic, anti-necrotic and anti-oxidant mechanisms	[100]
Human adipose tissue-derived MSCs (hADSCs)	Human adipose tissue	β-amyloid peptide	We found that hADSCs secrete exosomes carrying enzymatically active neprilysin, the most important β-amyloid peptide (Aβ)-degrading enzyme in the brain	[101]
Platelet-extracellular vesicles (PEVs)	Platelet	SH-SY5Y cells/Dopaminergic neurons	PEVs aided in the restoration of neuronal functions in SH-SY5Y cells and demonstrated remarkable neuroprotective capabilities against erastin-induced ferroptosis in dopaminergic neurons. In microglial cells, they promoted anti-inflammatory responses, particularly under inflammatory conditions	[102]
Exosome-like nanovesicles from citrus lemon (EXO-CLs)	Citrus lemon	SH-SY5Y cells	EXO-CLs demonstrated permeability across the BBB and displayed antioxidant activity comparable to ascorbic acid	[103]
Circulating small extracellular vesicles (sEVs) from AD patients	Circulating small extracellular vesicles (sEVs) from AD patients	BACE1	miR-342-5p enrichment in Exo-APP ameliorated amyloid pathology in the recipient cells	[105]
HBMVECs-Ex	Human brain microvascular endothelial cells	Amyloid-β	HBMVECs-Ex inheriting P-gp greatly facilitated the cerebral clearance of Aβ by effectively transporting Aβ out of brain and potently ameliorated cognitive dysfunction in AD mice	[106]

### Mesenchymal stem cells-derived exosomes

Mesenchymal stem cells (MSCs) are adult stem cells that can be isolated from connective tissues (including bone marrow and adipose tissue) and have become attractive candidates for cellular therapy applications. Exosomes derived from mesenchymal stem cells (MSCs-exos) possess immunomodulatory, neuroprotective effect, neurodegenerative promotion and abnormal protein clearance effects [95]. Currently MSCs-exos are increasingly being studied in various AD models, indicating that they may be feasible therapeutic agents for treating various diseases (as summarized in Table 1) [96]. For example, Hou et al. found that MSCs-exo could treat AD by promoting Aβ degradation, regulating immune responses, protecting neurons, promoting axon growth and improving cognitive impairment [97]. Besides, Cui et al. discovered that exosomes from hypoxia-preconditioned MSCs could improve cognitive decline in APP/PS1 mice by rescuing synaptic dysfunction and regulating inflammatory responses, with the potential mechanism being a significant increase in miR-21 expression in MSCs after hypoxia treatment [98]. Additionally, exosomes from human umbilical cord mesenchymal stem cells (hucMSCs-exos) [98], exosomes from bone marrow mesenchymal stem cells (BMSC-exos) (Fig. 5C) [89] and exosomes from early-passage rat bone marrow mesenchymal stem cells (BM-MSC-exos) [100] could also reduce neuronal cell damage in AD and exert therapeutic effects. Moreover, Katsuda et al. also found that human adipose tissue-derived MSCs (shADSCs) could secrete exosomes carrying active neuropeptides, which could degrade Aβ and potentially treat AD [101].

### Other cells-derived exosomes

**Fig. 6:**
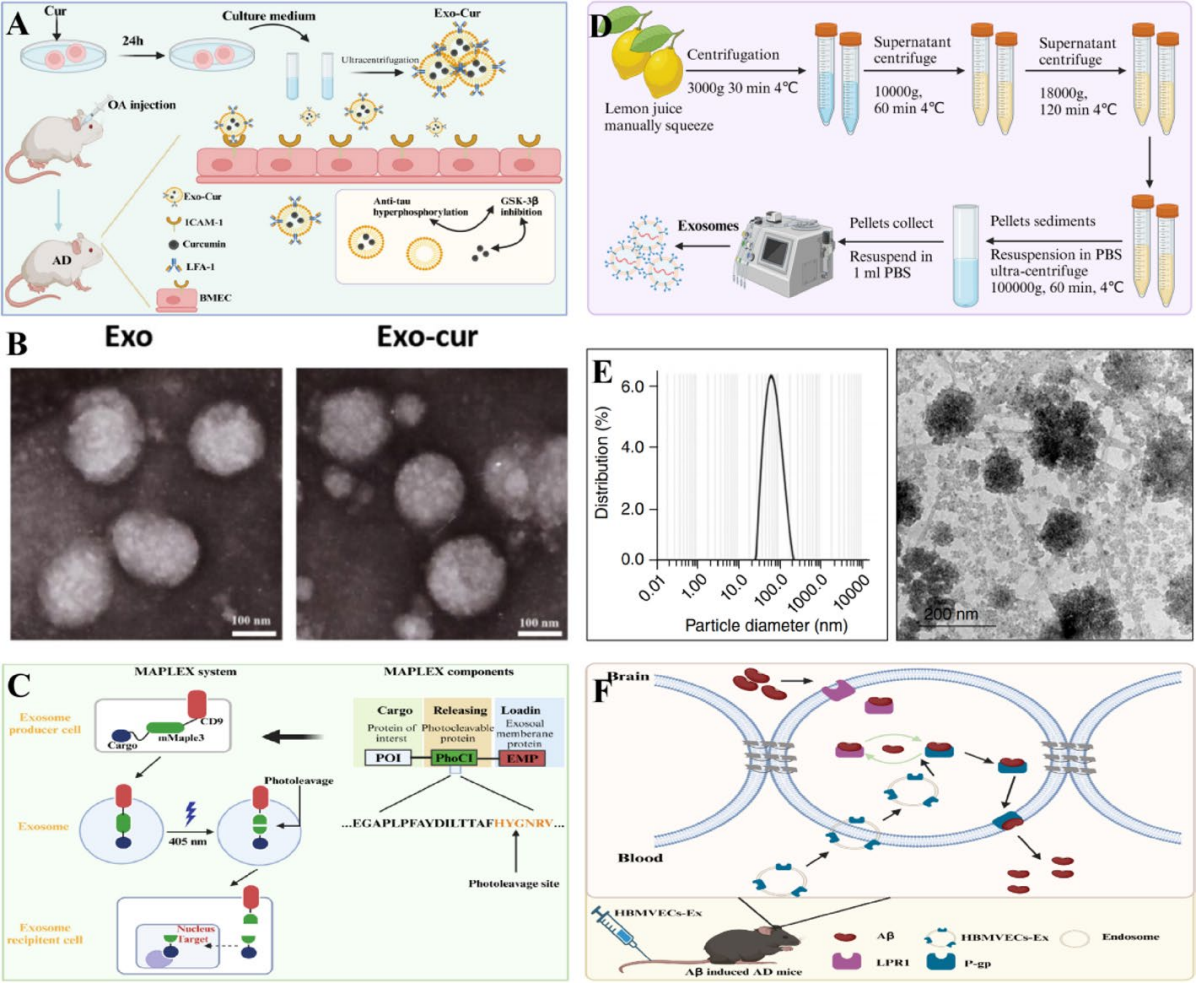
The primary hypothesis of study. Exosomes drived from curcumin-treated (primed) cells (Exo-cur) can better relive the symptoms of AD by inhibiting phosphorylation of Tau protein through AKT/GSK-3β pathway [108] (**A**); Morphology of Exo and Exo-cur observed by TEM [108] (**B**); Schematic design of the MAPleX system. (right) The MAPleX system consists of three components: a cargo protein, a Phocl, and an exosomal membrane protein (eMP). (left) mMaple3 is the Phocl, and cd9 is the eMP. cd9 enables cargo loading into the exosomes. once the exosome has been isolated, 405-nm light is used to induce photocleavage of mMaple3, which releases the cargo protein from the exosomal membrane. The 405-nm light-illuminated exosomes can then be purified and deliver functional cargo protein to the recipient cell [115] (**C**); Schematic diagram of EXO-CL separation by filtration and ultracentrifugation [103] (**D**); Determination of mean particle size of developed EXO-CL and TEM image of developed EXO-CL [103] (**E**); Schematic diagram of brain microvascular endothelial cell-derived exosomes enhancing Aβ clearance by up-regulating P-gp and improving cognitive dysfunction in AD mouse models [106] (**F**)

Furthermore, in addition to the common sources of exosomes, various other sources of exosomes have been found to be useful for the treatment of AD (as summarized in Table 1). For example, PEVs isolated from clinical-grade PCs of healthy donors using high-speed centrifugation could help restore neuronal function in SH-SY5Y cells and showed significant neuroprotective effects against erastin-induced ferroptosis in dopaminergic neurons (Fig. 5D) [102]. Besides, exosome-like nanovesicles from lemons (EXO-CLs) could penetrate the BBB and exhibited antioxidant activity comparable to ascorbic acid without affecting cell viability (Fig. 6D, E) [103]. In addition, human-induced GABAergic progenitor cells improved cognitive function in mice and inhibited astrocyte activation through anti-inflammatory exosomes, with the mechanism related to the regulation of the tumor necrosis factor (TNF) pathway mediated by CD4 + Th1 cells [104]. Then, circulating small cell-derived miR-342-5p targeted β-site APP cleaving enzyme 1 to improve β-amyloid formation in AD [105]. Also, brain microvascular endothelial cell-derived exosomes effectively improved cognitive dysfunction in a mouse AD model by upregulating P-gp, thereby enhancing Aβ clearance [106]. Additionally, plant sphingolipids could promote exosome release and alleviate amyloid β pathology in an AD mouse model [107].

In summary, exosomes derived from stem cells and other cell types holds great promise for the treatment of AD, but more research is needed to optimize treatment regimens and ensure their safety and efficacy (Fig. 6F) [106]. Besides, although the low immunogenicity and ease of acquisition of stem cell-derived exosomes have made them particularly attention-grabbing, their detailed therapeutic mechanisms are not yet fully understood and require further investigation.

## Bioengineered exosomes for the treatment of AD

The bio-synthetic exosome therapy for AD is an emerging regenerative medicine approach and it has received extensive attention in scientific research and clinical trials in recent years. Studies have shown that exosome-based therapies exhibit low immunogenicity and inflammatory responses [107]. Moreover, biosynthetic exosomes can carry selected drug molecules, such as nerve growth factors, anti-inflammatory factors and small molecule drugs to reduce the accumulation of abnormal proteins or promote the regeneration and repair of neural cells.

### Nanodrug loading strategies based on exosomes

Recent research has demonstrated that exosomes can serve as drug delivery carriers to penetrate BBB and target drug delivery to lesion sites for therapeutic effects (as summarized in Table 2). For example, exosomes pretreated with piperine (Exo-cur) designed by Wang et al. could efficiently cross BBB through receptor-mediated transcytosis, enter the brain tissue and inhibit Tau phosphorylation to improve targeted drug delivery and restore neuronal function in AD (Fig. 6A, B) [108]. Zhao et al. constructed exosomes loaded with Ber and Pal that could promote drug accumulation in cells and tissues, improve the targeting of drugs across BBB and inhibit Aβ plaque formation and microglial activation, while regulate the secretion of inflammatory factors [109]. Additionally, exosomes loaded with Olesoxime-resveratrol (OLX-RSV) could inhibit Aβ1-42 aggregation and cross BBB, showing good biocompatibility [110]. In addition to loading herbal extracts, therapeutic nucleic acids (miRNA-22) and proteins (Tom40 protein) can also be loaded into exosomes for the treatment of AD [111, 112]. In summary, exosome-based nanomedicine delivery strategies have shown promising prospects, but more in-depth mechanism studies and clinical studies are needed to promote their clinical application.

**Table 2:** Summary of sources, therapeutic targets and molecular mechanisms of bioengineered exosomes

Name	Medicine	Therapeutic targets	Molecular mechanisms	References
Exosomes derived from curcumin-treated (primed) cells (Exo-cur)	Curcumin	LFA-1 and ICAM-1	Exosomes derived from curcumin-treated (primed) cells (Exo-cur) can better prevent the death of neurons in vitro and in vivo to relieve the symptoms of AD by inhibiting phosphorylation of the Tau protein through activating the AKT/GSK-3β pathway	[108]
Exos-Ber/Pal	Ber/Pal	Microglial cells	Ber and Pal (Ber/Pal) modulated microglial inflammatory cytokine levels	[109]
Olesoxime-Resveratrol (OLX-RSV) encapsulated in exosomes	Olesoxime	Human neuroblastoma cells	Exosomes loaded with Olesoxime significantly enhanced the learning and memory of spatial cues in APP/PS1 mice	[110]
Tom40 engineered exosomes	Tom40	Mitochondrial	Tom40 protein delivery by the exosome successfully protected the cells against hydrogen peroxide-induced oxidative stress	[111]
miRNA-22 loaded exosomes (Exo-miRNA-22)	miRNA-22	Microglia	miRNA-22-loaded ADMSC-derived exosomes could decrease the release of inflammatory factors by inhibiting pyroptosis, thereby playing a synergetic therapeutic role with exosomes on AD	[112]
Mannose-modified exosomes laden with Gem (MExo-Gem)	Gem	Microglia	Exosomal Gem activated lysosomal activity and accelerated lysosome-mediated clearance of Aβ in microglia	[113]
Engineered activated neutrophil-derived exosomes (MP@Cur-MExo)	Curcumin	Mitochondrial	MP@Cur-MExo restored mitochondrial function and reduced Aβ-induced mitochondrial damage, thereby attenuating AD progression	[114]
mMaple3-mediated protein loading into and release from exosome (MAPLEX)	mMaple3-mediated protein	β-site amyloid precursor protein cleaving enzyme 1	This approach led to a significant reduction in Bace1 expression, improved recognition memory impairment, and reduced amyloid pathology in 5xFAD and 3xTg-AD mice	[115]
TSEL	siBACE1 /pTREM2	TREM2	The in vivo study suggests that TSEL through the synergistic effect of two gene drugs can ameliorate APP/PS1 mice cognitive impairment by regulating the activated microglial phenotype, reducing the accumulation of Aβ, and preventing the retriggering of neuroinflammation	[116]
Lesion-recognizing nanoparticles	BACE1 siRNA/caspase-3 siRNA	BACE1/caspase-3	The cores of NPs directly enter into the cytoplasm and achieve the controlled release of siRNAs in a high-ROS environment to downregulate the level of BACE1 and caspase-3 to ameliorate neurologic injury	[117]
Fe65-EXO-Cory-B	Corynoxin-b	Interaction between Fe65 and APP	The Fe65-engineered HT22 hippocampus neuron cell-derived exosomes (Fe65-EXO) loaded with Cory-B (Fe65-EXO-Cory-B) hijacked the signaling and blocked the natural interaction between Fe65 and APP, enabling APP-targeted delivery of Cory-B	[118]
RVG peptide-exo	RVG peptide	α7-nAChR	When incubated with Aβ-producing N2a cells, it significantly decreased intracellular and secreted Aβ40 levels, a potency that is superior to exosomes derived from adipose-derived stem cell	[119]

### Targeted precision delivery strategies based on exosomes

**Fig. 7:**
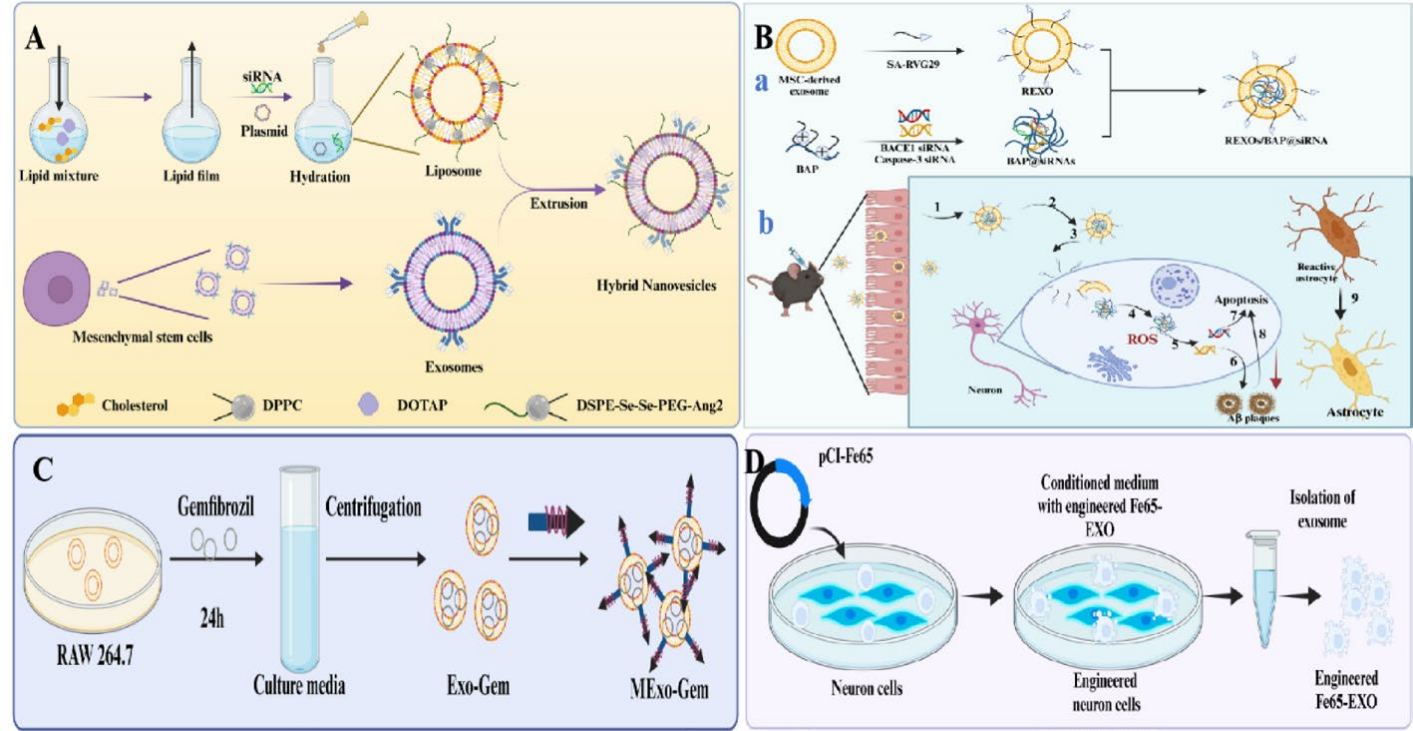
Schematic Illustration of the Synthesis Process of Exosome-Liposome Hybrid Nanovesicles [116] (**A**). Schematic Illustration of the Lesion-Recognizing NPs. (a) Preparation of the Lesion-Recognizing NPs. (b) Mechanism of the Lesion-Recognizing NPs for the Synergistic Treatment of AD. After intranasal administration, (1) the lesion-recognizing NPs crossed the nasal mucosa and (2) migrated to the diseased brain area. (3) The NPs recognized the neurons and (4) fused with the cell membrane of neurons to directly release the cores of NPs into the cytoplasm. (5) The cores of NPs achieved the controlled release of siRNAs in the high-ROS environment to downregulate the level of BACE1 and caspase-3 to (6) reduce the Aβ plaques and (7, 8) inhibit the apoptosis of neurons. (9) The MSC-derived exosomes reduced the number of reactive astrocytes for the synergistic treatment of AD [117] (**B**). Schematic diagram of preparation of bioengineered microglia targeting exosomes [113] (**C**). Schematic diagram showing the steps involved in the production of engineered Fe65-EXO [118] (**D**)

To further improve the targeted delivery performance of exosomes, researchers have developed various targeted receptor/ligand-modified exosome delivery systems. For example, manganese was modified onto exosomes (MExo-Gem) to target the delivery of gemfibrozil (Gem) and restore the lysosomal activity of microglia for clearing Aβ aggregates. Mechanistically, mannose-modified exosomes can interact with the mannose receptor on microglia, leading to the uptake of exosomes by microglia (Fig. 7E) [113]. For example, Zhang et al. constructed neutrophil-derived exosomes (MP@Cur-MExo) with surface-modified magnetic nanoparticles (SPIONs) that targeted mitochondria and Aβ, which could alleviate Aβ-induced neurotoxicity and improve mitochondrial function in neurons [114]. Furthermore, photo-cleavable proteins (mMaple3), rabies virus glycoprotein peptides, RVG peptides, Fe65 protein, angiopep-2 peptides and others have been used to modify the surface of exosomes to increase targeting and have shown promising therapeutic effects in AD (Figs. 6C, 7A–D) [115–119]. In general, the above reports on biosynthetic exosomes provide new insights into the treatment of AD and further confirm the potential of exosome-based nanotherapeutic strategies. However, these studies are still in the stage of theoretical verification. How to effectively produce biosynthetic exosomes on a large scale and how to precisely control the molecular composition and function of exosomes are the main challenges of current research.

## Conclusions

The limitations of existing treatment methods for AD have driven the research on innovative therapies based on nanotechnology. Exosomes, as a natural biological carrier, have become a research hotspot in the field of AD treatment. Their core advantages lie in: (1) Targeted delivery capability: Exosomes can penetrate the BBB to achieve precise drug delivery; (2) Multifunctionality: Through surface modification or content recombination, different therapeutic strategies targeting different targets can be developed; (3) Diversity of cell sources: Various sources of exosomes have shown potential in improving cognitive function.

However, the clinical translation of exosome therapy still faces multiple challenges: (1) The technology for large-scale production still needs to be developed and optimized; (2) The targeting specificity of existing modification strategies in the complex brain microenvironment needs to be improved; (3) Long-term biocompatibility and other aspects still require systematic verification. We believe that future research will focus on: developing efficient and stable exosome engineering technologies; establishing multi-targeted combined treatment strategies; and deepening mechanism analysis through preclinical models (such as organoids). In summary, current research shows that exosome therapy provides a new paradigm for the treatment of AD, but its clinical implementation urgently requires interdisciplinary collaboration to break through technical bottlenecks and establish standardized evaluation systems.

## Data Availability

All data generated or analysed during this study are included in this published.

## Abbreviations

AD: Alzheimer's disease
Aβ: Amyloid beta proteins
APP: Amyloid precursor protein
ATP: Adenosine triphosphate
BBB: Blood-brain barrier
CNS: Central nervous system
ESEs: Early sorting endosomes
BMSC-exos: Exosomes from bone marrow mesenchymal stem cells
BM-MSC-exos: Exosomes from early-passage rat bone marrow mesenchymal stem cells
hucMSCs-exos: Exosomes from human umbilical cord mesenchymal stem cells
MSCs-exos: Exosomes from mesenchymal stem cells
EXO-CLs: Exosome-like nanovesicles from lemons
shADSCs: Human adipose tissue-derived MSCs
hUSSC: Human non-restrictive somatic stem cell
H_2_O_2_: Hydrogen peroxide
OH^·^: Hydroxyl radical
ILVs: Intraluminal vesicles
IL-1β: Interleukin-1β
LSEs: Late sorting endosomes
MACs: Membrane attack complexes
MSCs: Mesenchymal stem cells
mtDNA: Mitochondrial DNA
MVBs: Multivesicular bodies
NPs: Nanoparticles
NDs: Neurodevelopmental disorders
ACh: Neurotransmitter acetylcholine
OLX-RSV: Olesoxime-resveratrol
PHFs: Paired helical filaments
ROS: Reactive oxygen species
SNARE: Soluble N-ethylmaleimide-sensitive factor attachment receptor
NSC-exos: Stem cells-derived exosomes
O_2_-: Superoxide anion
SPIONs: Surface-modified magnetic nanoparticles
TNF: Tumor necrosis factor
TNFα: Tumor necrosis factor α
BACE1: β-Secretase
MOFs: Metal-organic frameworks
NPs: Nano-polymers
MAPT: Microtubule-associated protein tau
BACE1: β-Secretase
AICD: An intracellular domain
Alix: ALG-2 interacting protein X‌
TSG101: Tumor susceptibility gene 101 protein
SNARE: Soluble NSF attachment protein receptor
RAB GTPases: Ras-related protein in brain GTPases
M2-EXOs: M2-polarized microglia
PINK1: PTEN-induced putative kinase 1
hUSSC: Human non-restrictive somatic stem cell
NMDAR1: N-methyl-D-aspartate receptor subunit 1
PPKα: Protein kinase C alpha
Exo-cur: Exosomes pretreated with piperine
Gem: Gemfibrozil
MExo-Gem: Manganese was modified onto exosomes
SPIONs: Surface-modified magnetic nanoparticles
